# Suspension of the penis – dissection, anatomical description and highlighting of anatomical risks in sectioning the suspensory ligaments

**DOI:** 10.1186/s12610-023-00202-1

**Published:** 2023-10-24

**Authors:** Florin-Mihail Filipoiu, Radu-Tudor Ion, Zoran Florin Filipoiu, Adrian-Daniel Tulin, Octavian Enciu, Mihaly Enyedi

**Affiliations:** 1 Morphological Sciences Department, Anatomy Discipline, University of Medicine and Pharmacy “Carol Davila” Bucharest, Bucharest, Romania; 2 Doctoral School of the University of Medicine and Pharmacy “Carol Davila” Bucharest, Eroii Sanitari Blvd, no.8, Bucharest, 050474 Romania; 3 Surgery Department, University of Medicine and Pharmacy “Carol Davila” Bucharest, Bucharest, Romania

**Keywords:** Suspensory ligament of the penis, Fundiform ligament of the penis, Penis augmentation, Dissection, Ligament suspenseur du pénis, Ligament fundiforme du pénis, Augmentation du pénis, Dissection

## Abstract

**Background:**

The suspension of the penis is provided by two ligaments: fundiform and suspensory. These ligaments are sectioned during some augmentative surgical procedures. The structure, the relations and the variability of these ligaments have been demonstrated. The penile neurovascular bundle and its relationships have also been emphasized. A clear knowledge of these details should ensure a reduction of the risk of surgical injury during augmentation procedures.

**Results:**

We dissected the ligaments providing the suspension of the penis in 7 formalized corpses. We identified, for each of the ligaments, the origin, the insertion and the relations. The dissection pieces were photographed and the images obtained were discussed upon. We described the variability of the anatomical distribution and highlighted the relations with the vascular and nervous structures for each of these ligaments. The anatomical variability of the fascia and the relations with the base of the penis were also emphasized. For the suspensory ligament, we identified three groups of fibers through which it is attached to the penile body.

**Conclusions:**

The dissections were conducted in layers, corresponding to the operative steps for the penile augmentation procedures. We believe that our study highlights the anatomical basis necessary to safely perform these surgeries. The study contributes to the description of the anatomical variability of the ligaments and logically presents details that contribute to preventing most surgical incidents.

## Résumé

**Contexte:**

La suspension du pénis est assurée par deux ligaments: fundiforme et suspenseur. Ces ligaments sont sectionnés lors de certaines interventions chirurgicales d’agrandissement pénien. La structure, les relations, et la variabilité de ces ligaments ont été démontrées. Le faisceau neurovasculaire pénien et ses relations ont également été soulignés. Une connaissance claire de ces détails devrait assurer une réduction du risque de blessure chirurgicale pendant les procédures d'augmentation.

**Résultats:**

Nous avons disséqué les ligaments assurant la suspension du pénis sur 7 cadavres standardisés. Nous avons identifié, pour chacun des ligaments, l'origine, l'insertion et les relations. Les pièces de dissection ont été photographiées et les images obtenues ont été discutées. Nous avons décrit la variabilité de la distribution anatomique, et mis en évidence les relations avec les structures vasculaires et nerveuses pour chacun de ces ligaments. La variabilité anatomique du fascia et les relations avec la base du pénis ont également été soulignées. Pour le ligament suspenseur, nous avons identifié trois groupes de fibres par lesquelles il est attaché au corps pénien.

**Conclusions:**

Les dissections ont été effectuées en couches, correspondant aux étapes opératoires des procédures d'augmentation du pénis. Nous pensons que notre étude met en évidence la base anatomique nécessaire pour effectuer ces chirurgies en toute sécurité. L'étude contribue à la description de la variabilité anatomique des ligaments et présente logiquement des détails qui contribuent à prévenir la plupart des incidents chirurgicaux.

## Introduction

There are two ligaments that contribute to the maintaining of the physiological prepubic curvature of the penis: a superficial one, known as the fundiform ligament, and a deeper one, known as the suspensory ligament [1]. These ligaments usually connect the penile body to the pelvis, so that the penile body is maintained on the midline and it takes a position of approximately 30 degrees from the anterior abdominal wall, so it allows coitus to occur [2] and the movements of the penis to follow the movements of the pelvis [1, 3]. R. F. Kropman, P. L. Venema and R. C. M. Pelger [4] state that traumatic rupture of the suspensory ligament leads to the hypermobility of the penis, which tends to slip out of the vagina during intercourse, a situation that also occurs in the congenital absence of the ligament [2].

In the context of a considerable amount of social pressure that links sexual performance to the size of the penis, penile lengthening surgeries have been proposed to men with true congenital microphallus, which is defined as a penis with normal anatomy, but with a size less than 2.5 SD below the normal population [5]. These penile lengthening procedures may also be performed in the case of conditions that lead to penile shortening: radical prostatectomy in prostate cancer, Peyronie’s disease [6] or conditions that lead to buried penis, most commonly obesity or post-circumcision scar formation [7]. The popularity of penile length or girth augmentation is proven by the fact that in US alone, 10.000 men underwent these procedures between 1991 and 1998 [8]. There are many types of procedures that have been described in the literature [5], but there is no consensus regarding the best surgery technique or the optimal moment of undertaking such invasive procedures, if ever [9].

Littara et al. [10] stated that, out of 355 performed surgical procedures, 21 were represented by cosmetic elongation, 33 of them were enlargement surgeries and 301 of them were comprised of combined elongation and enlargement procedures.

The surgical lengthening technique mainly involves an inverted "V–Y" penopubic skin advancement [11], with identification and successive sectioning or division of the penile ligaments [3], sometimes in combination with the removal of suprapubic fat tissue [12]. The use of postoperative penile weights is prescribed by most surgeons [11].

The procedure might require the addition of a silicone spacer placed between the penis and the pubis, mainly to prevent the ligaments from reattaching [13]. The surgical procedures are prone to complications, mainly cosmetic penile deformities, impotence, incontinence or persistent penile pain [14].

In the literature, however, we have encountered terminology that can often be confusing. For example, in “Traité d'Anatomie humaine—par L. Testut” only the suspensory ligament is described, while other publications such as “Gray's Anatomy – The Anatomical Basis of Clinical Practice 41st Edition” do not mention the fundiform ligament. In other articles, the term fascia Scarpa is used to describe the fundiform ligament [10, 15]. On the other hand, we consider that many studies regarding this topic lack a detailed anatomical description of these ligaments, especially when describing the arrangement of the connective tissue bundles [2, 11].

In this study, we aim to identify these ligaments by dissection, to describe the important vascular and nervous relations and to demonstrate the distribution of the different structural bundles.

## Material and methods

The dissections were performed on seven cadavers in the laboratory of the Anatomy Department of the “Carol Davila” University of Medicine and Pharmacy.

The cadavers have been previously formalized by injection into the femoral artery with a 10% formalin solution and then kept for 30 days in tanks containing the same concentration of formalin.

The dissections were performed in anatomical layers. The fundiform and suspensory ligaments were identified and the origin, insertion, dimensions and relations with the penile sheaths were described. The dissection pieces were photographed and discussed upon. The images have been digitally edited without altering the scientific content.

## Results and discussion

To identify the fundiform ligament, we sectioned and then removed the skin and the superficial abdominal fascia.

We have identified three types of fundiform ligament:

1. A unique triangular structure;
2. A double structure accompanied by accessory fascicles;
3. A ligament structure with the aspect of an undifferentiated fibrous connective band.

In four cadavers, the fundiform ligament was identified in the form of a unique triangular-looking fibrous structure, with the superior angle attached to the abdominal linea alba. Its length was between 5–7 cm, measured from the dorsal aspect of the penis to the apex of the ligament. Its width at the base is between 2–3 cm.

**Fig. 1:**
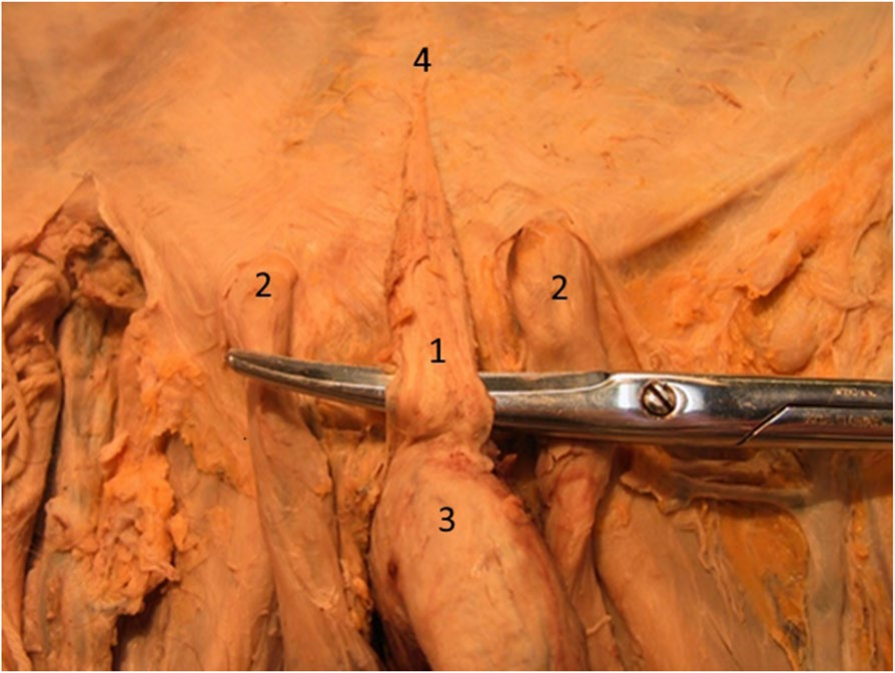
The fundiform ligament with triangular structure

Figure 1 shows the fundiform ligament, with a triangular aspect, between the superficial inguinal orifices, through which the spermatic cord passes.

In the upper part, the deep aspect of the ligament is attached to the abdominal linea alba. The ligament descends anteriorly from the pubic symphysis. The insertion allows a relatively easy separation of the ligament from the white line and its isolation by surgical dissection. Some of the fibers of the suspensory ligament are attached to the superficial fascia of the penis, while others surround the penile body. This arrangement explains the formation of the prepubic curvature of the penis. Sometimes this attachment can have a lower placement [16].

**Fig. 2:**
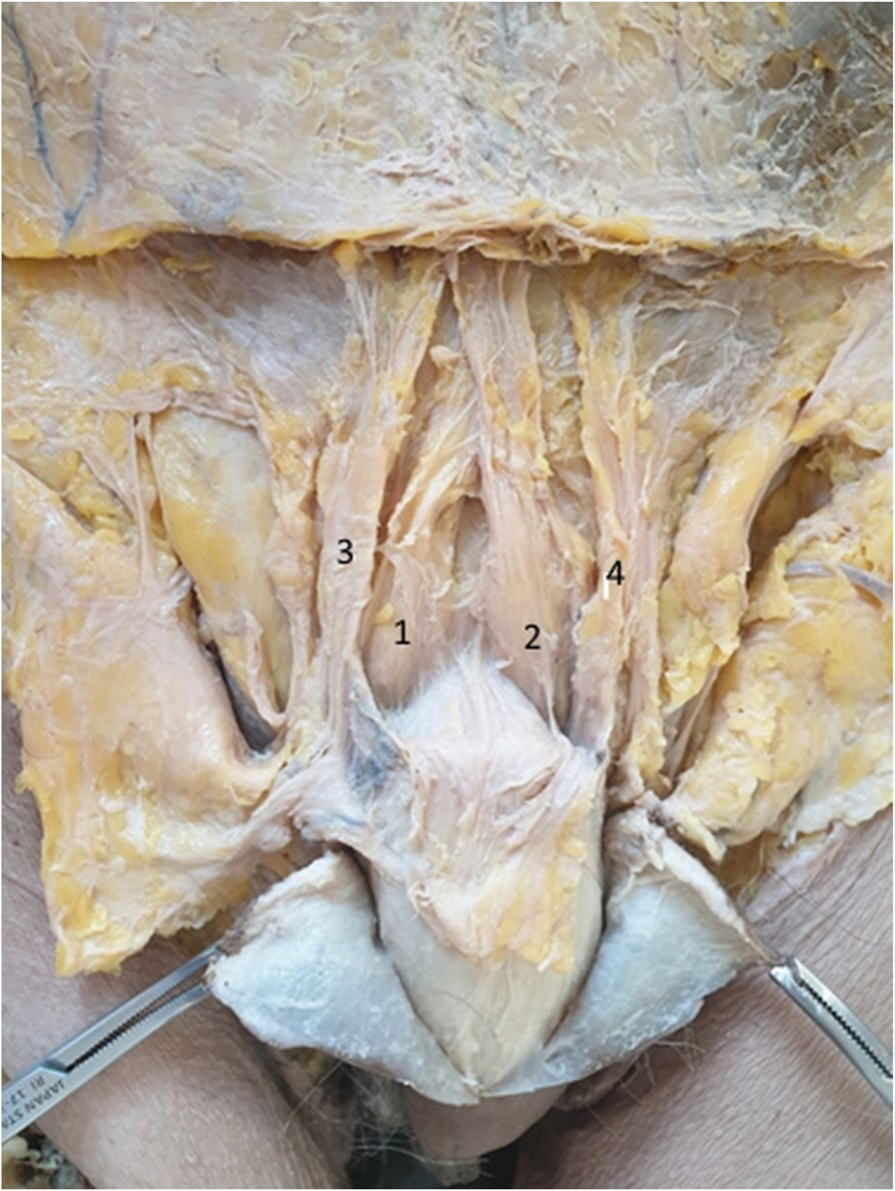
Double-structure fundiform ligament and accessory fascicles. 1. Right fundiform ligament. 2. Left fundiform ligament. 3,4. Right and left accessory fascicles

In one cadaver, we identified a fundiform ligament with a complex appearance. See Fig. 2.

Figure 2 shows the fundiform ligament, lying between the superficial fascia and the aponeurosis of the external oblique muscles. The ligament is comprised of two fibrous bundles which are spaced at the base and joined at the top, where it attaches to the white line [17].

This double structure must be taken into account when the surgeon intends to section the fundiform ligament. It is obvious that there may be a risk of sectioning a single ligament component. As can be seen in Fig. 2, the main ligament components are laterally doubled by fibrous bundles originating on the aponeurosis of the external oblique muscle and attached to the superficial fascia of the penis, anterior to the main ligamental structure. These fibrous bundles contain, at their insertion, the external pudendal vessels. The main bundles surround the penile body, which they support like a hammock, causing the prepubic curvature [13].

**Fig. 3:**
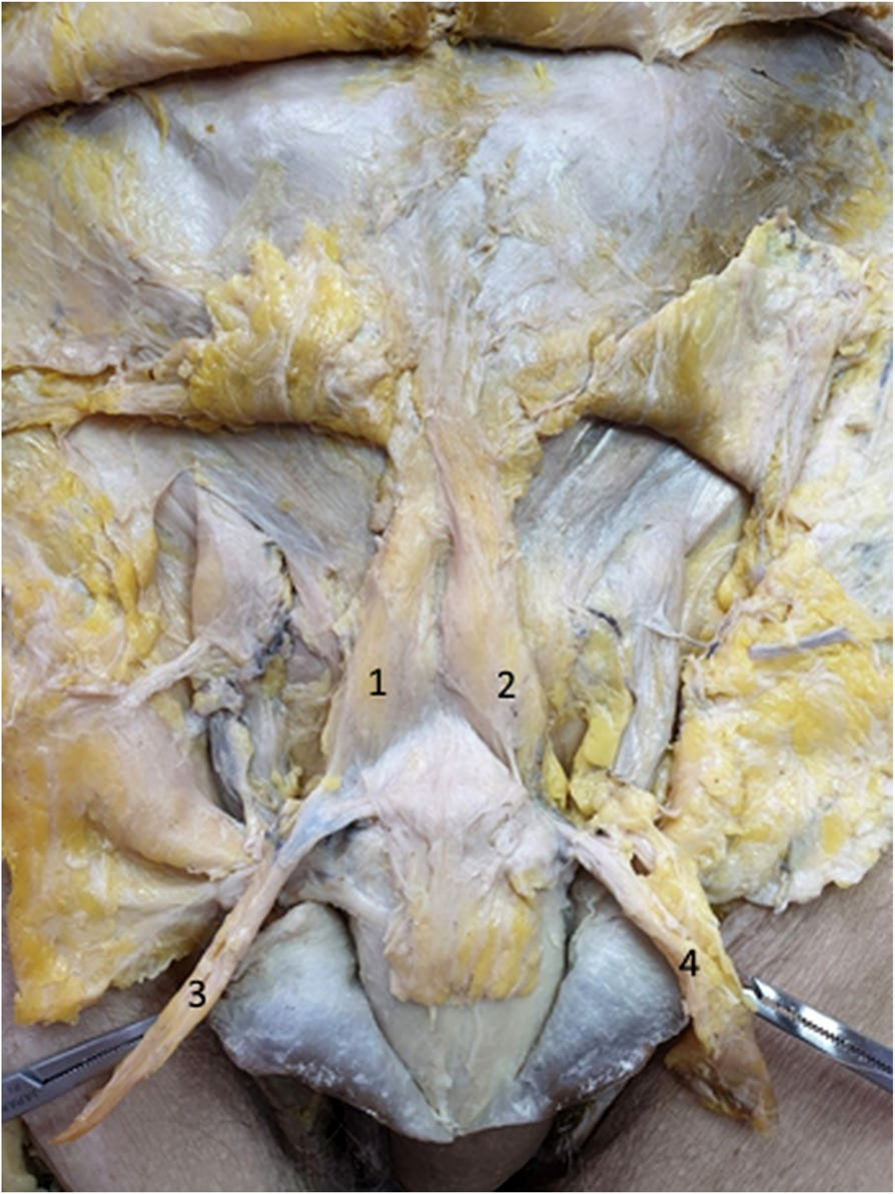
Double-structure fundiform ligament with accessory fascicles sectioned and folded. 1. Right fundiform ligament. 2. Left fundiform ligament. 3,4. Right and left sectioned accessory fascicles

The additional lateral ligament bundles were detached and folded down in the next step of the dissection of the cadaver depicted in Fig. 2. This step is shown in Fig. 3, where the main component, represented by a fundiform ligament with a double structure, remains in the center of the image.

**Fig. 4:**
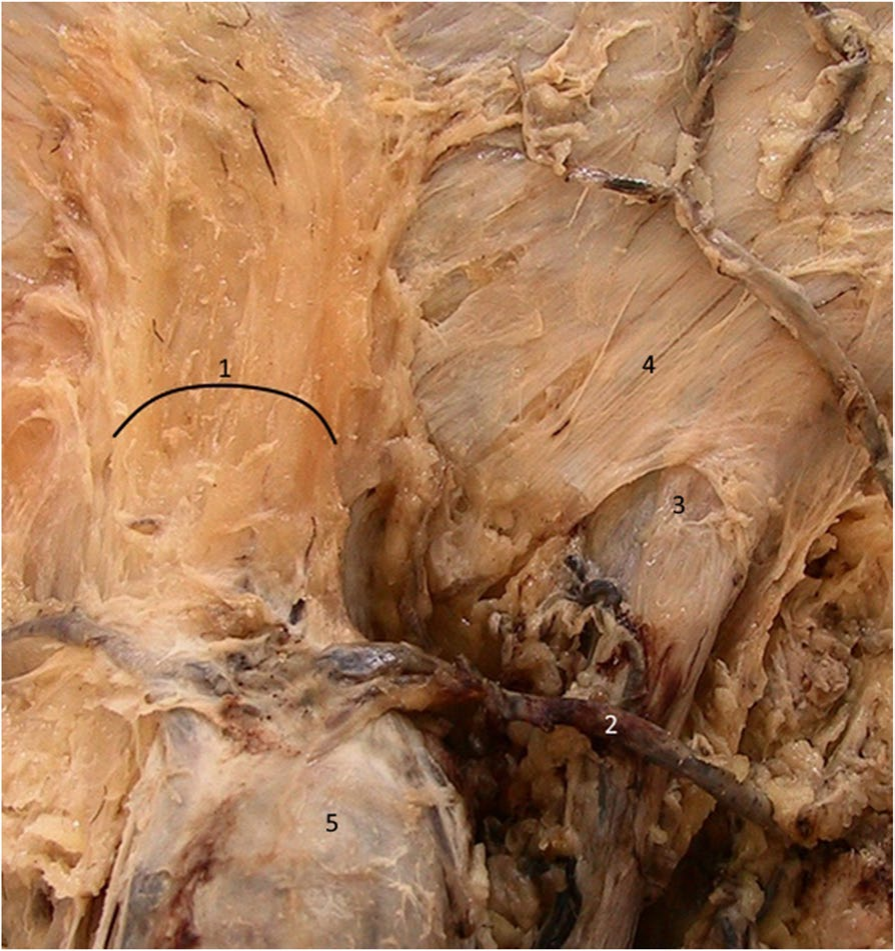
Ligament structure with the aspect of an undifferentiated connective-fibrous band. 1. Fundiform ligament. 2. External pudendal vessels

The third variant of fundiform ligament is presented in Fig. 4. In this situation, the ligament maintains its position, its situation and the relations to the penis but is not well-structured, being comprised of an undifferentiated bundle of fibers. The external pudendal vessels anastomose anteriorly to the lower part of the ligament. This relation is particularly important, because damage to these vessels without proper hemostasis can lead to the formation of a local hematoma, with the possibility of its intrapelvic migration [5, 11, 18].

**Fig. 5:**
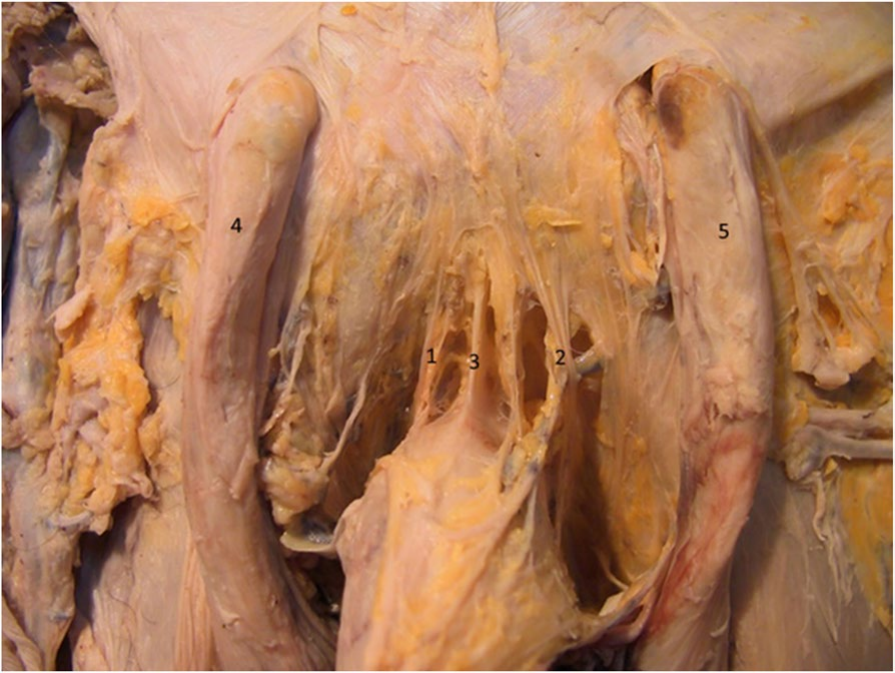
Relations of the fundiform ligament with the penis and the suspensory ligament. 1. Right bundle of the fundiform ligament. 2. Left bundle of the fundiform ligament. 3. Suspensory ligament. 4. Right spermatic cord. 5. Left spermatic cord

In the situation depicted in Fig. 5, we emphasized the fibers at the base of the fundiform ligament that diverge into two bundles, a right and a left one. Some of these fascial fibers penetrate the superficial fascia of the penis, while others surround the penile body to create the presymphyseal curvature. Some of the fibers of the fundiform ligament originate on the symphyseal arch and, usually, these fascicles contain the external pudendal vessels. At its base (close to the penis), the fundiform ligament is located superficially to the suspensory ligament of the penis [18].

**Fig. 6:**
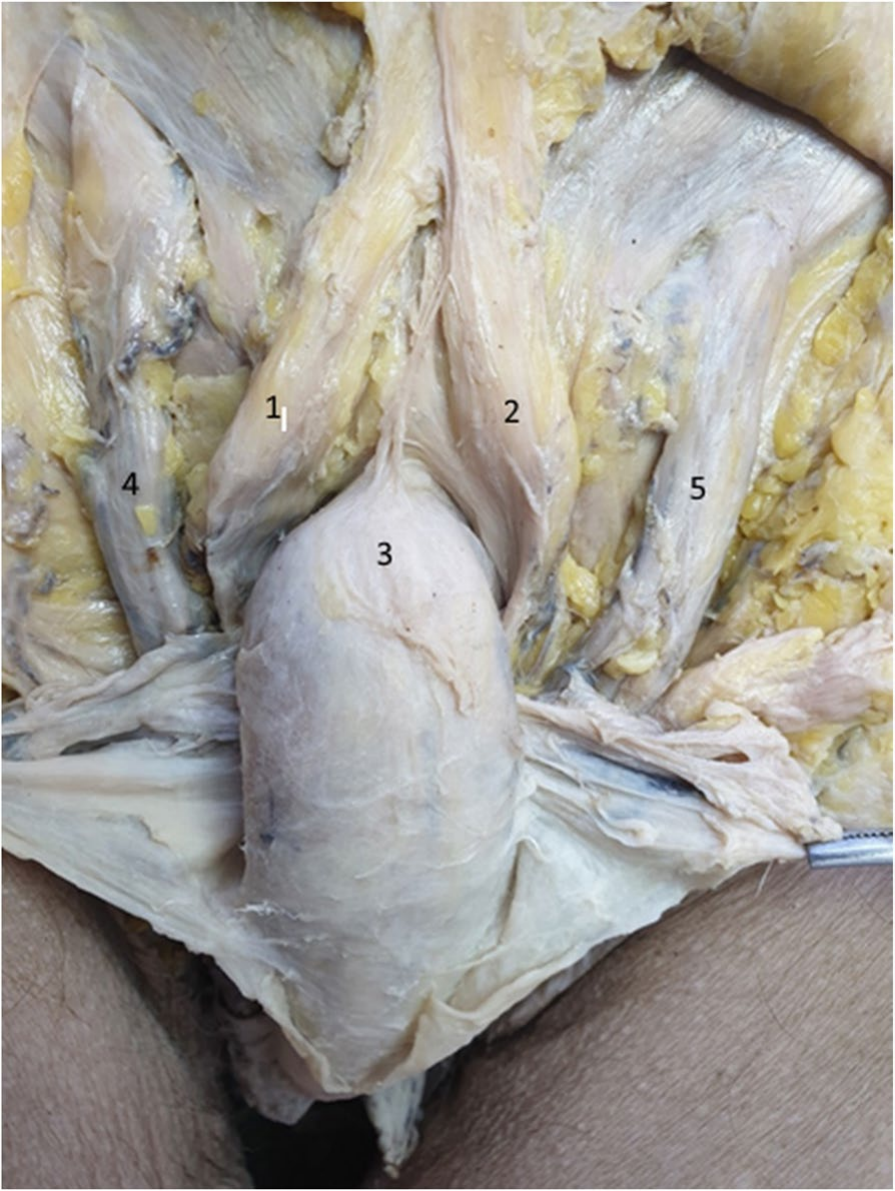
The fundiform ligament in relation to the penile body. 1. Right fascicle of the fundiform ligament. 2. Left fascicle of the fundiform ligament. 3. Body of the penis. 4. Right spermatic cord. 5. Left spermatic cord

**Fig. 7:**
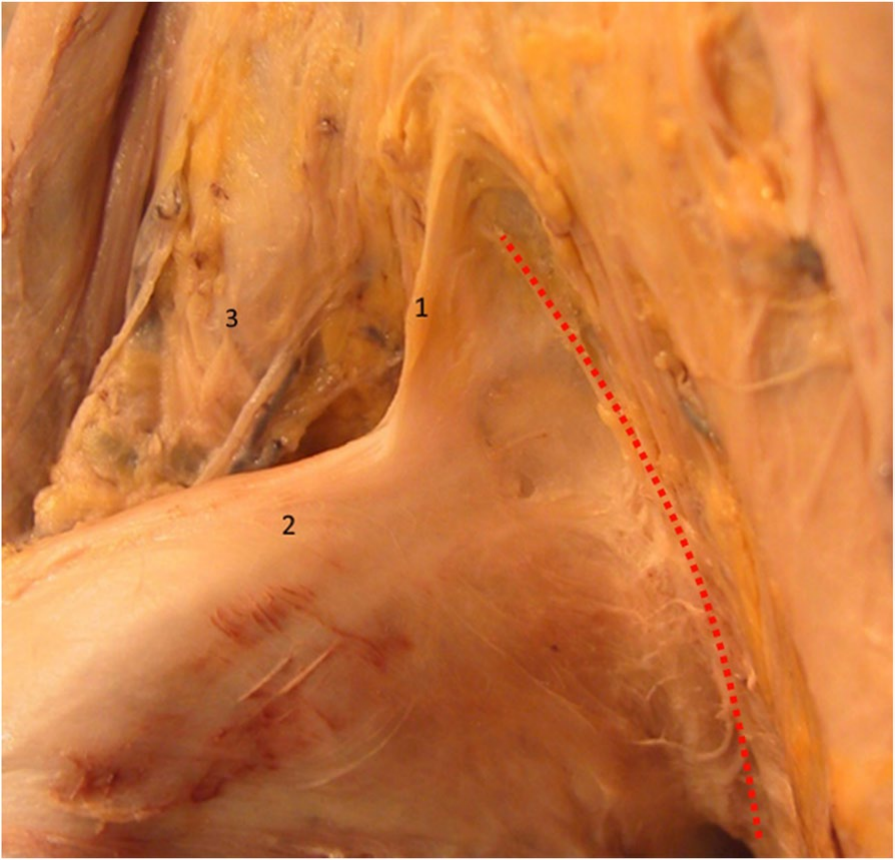
Highlighting the suspensory ligament. View from left lateral side. 1. Suspensory ligament of the penis. 2. Body of the penis. 3. Right spermatic cord. The red dotted line represents the Luschka ligament

In the dissection depicted in Figs. 6 and 7 we emphasized the fundiform ligament, structured as two separate fascicles. These well-individualized fascicles surround the penile body to create the presymphyseal curvature.

The suspensory ligament originates on the subsymphyseal angle and attaches to the dorsal aspect of the penis, deep to the fundiform ligament [14, 19].

It mainly consists of two laminas that fuse anteriorly in a unique border, creating the appearance of a triangular pyramid [4, 17]. There is a complex relationship between this structure and the penis. The fibers of the two lateral laminas surround the penis in relation to the deep penile fascia, strengthening the presymphyseal curvature of the penis [8, 11, 18].

**Fig. 8:**
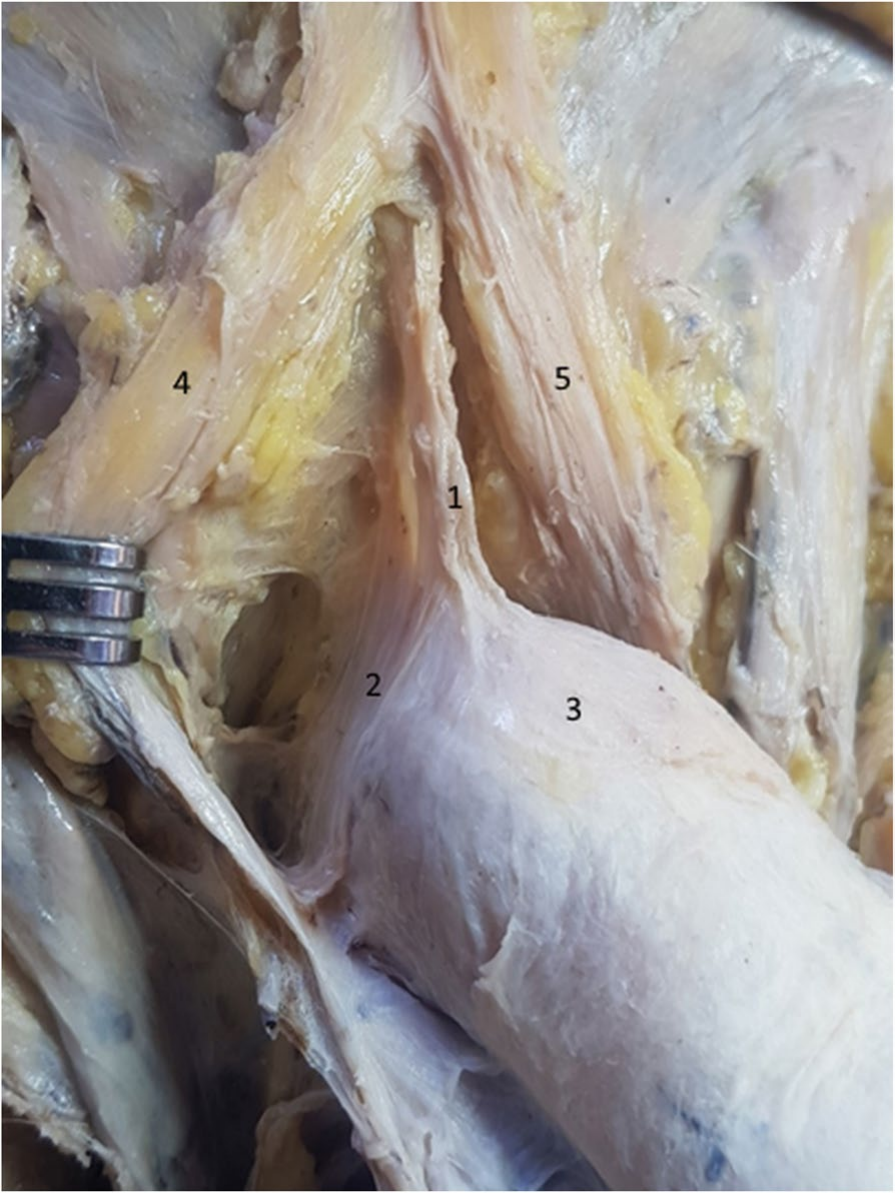
Hammock fibers of the suspensory ligament. 1. Suspensory ligament. 2. Hammock fibers of the suspensory ligament. 3. Body of the penis. 4. Right bundle of the fundiform ligament. 5. Left bundle of the fundiform ligament

Figure 8 clearly demonstrates the manner in which the fibers of the suspensory ligament surround the penile body. Similar fibers of the fundiform ligament are folded sideways. A mass of connective adipose tissue, located between the two ligaments, participates in closing the communication between the extrapelvic and intrapelvic regions. The communication with the intrapelvic space between the dorsal aspect of the penis and the symphyseal arch is closed by the fundiform ligament, the suspensory ligament and this connective adipose tissue known as the Luschka ligament [13, 15, 16].

**Fig. 9:**
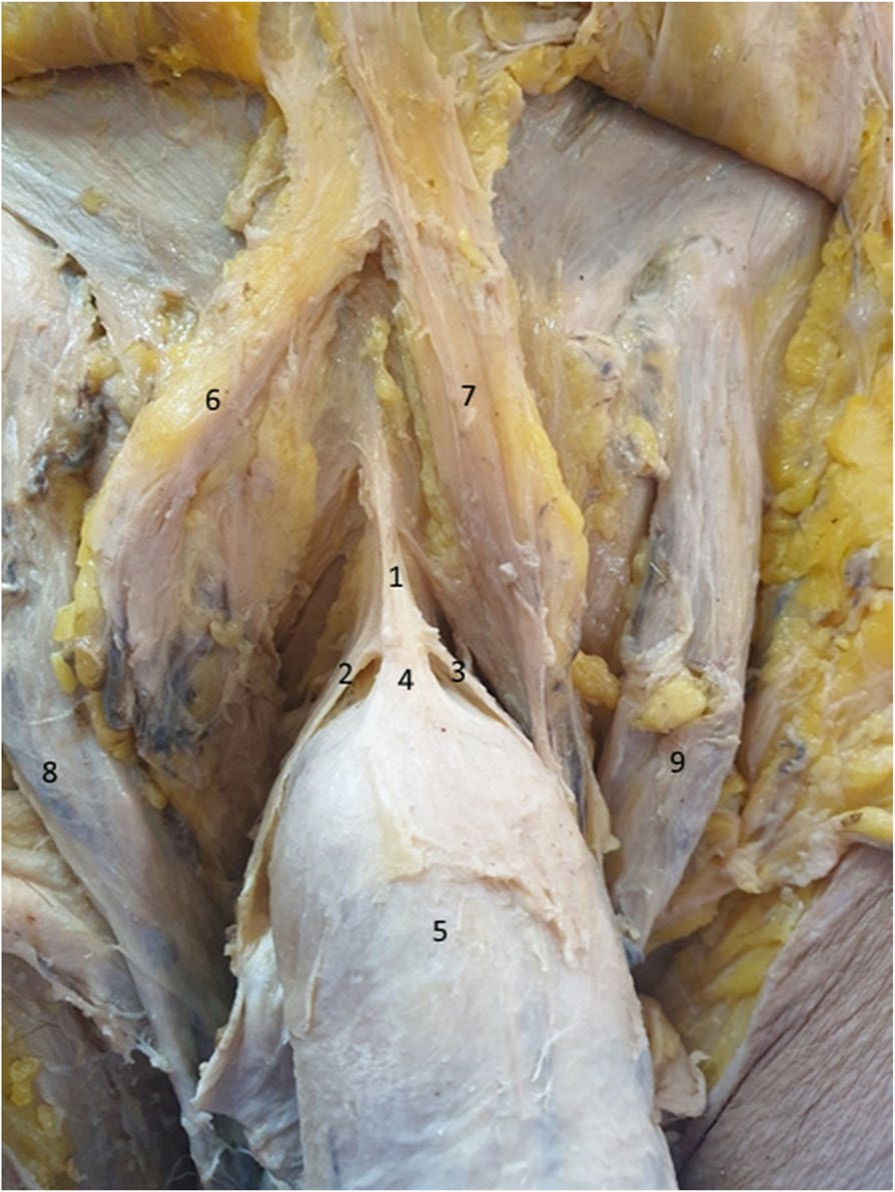
Suspensory ligament components. 1. Suspensory ligament. 2,3. Lateral bundles of the suspensory ligament. 4. Median bundle of the suspensory ligament. 5. Body of the penis. 6. Right fascicle of the fundiform ligament. 7. Left fascicle of the fundiform ligament. 8. Right spermatic cord. 9. Left spermatic cord

In Fig. 9, we emphasized the three bundles that attach the penis to the suspensory ligament. Two lateral bundles surround the penile body, while a median bundle is continued directly by the deep fascia of the penis. While the lateral bundles participate in the formation of the presymphyseal curvature, the median bundle maintains the penile body in the median sagittal position [2, 20].

**Fig. 10:**
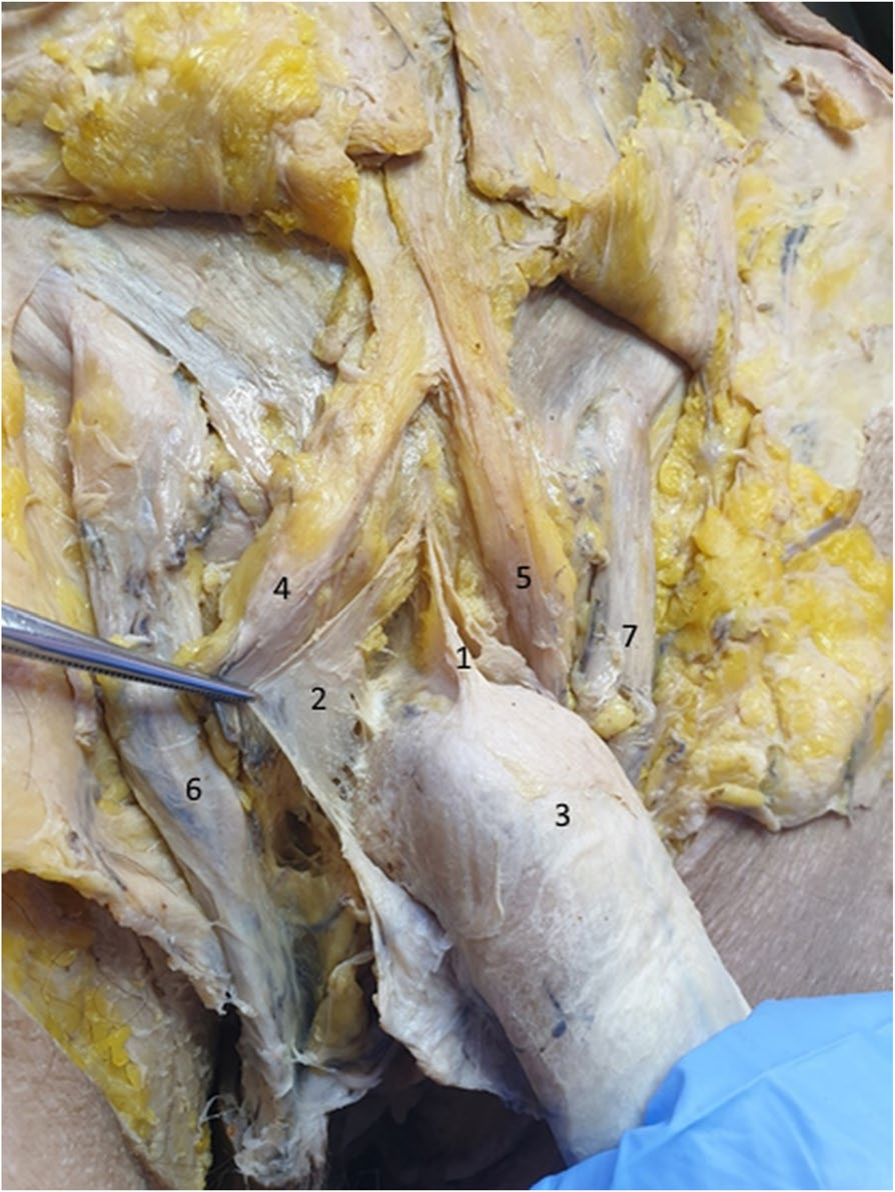
Suspensory ligament components – detail. The tweezer contains the right lamina of the suspensory ligament. 1. Median bundle of the suspensory ligament. 2. Right lateral bundle of the suspensory ligament. 3. Body of the penis. 4. Right fascicle of the fundiform ligament. 5. Left fascicle of the fundiform ligament. 6. Right spermatic cord. 7. Left spermatic cord

In Fig. 10, we separated the two lateral laminas of the suspensory ligament, demonstrating, once again, their circular route around the penis. However, the surprise comes from the median fiber group, which actually comprises a third ligament, with its inferior attachment continued by the deep fascia of the penis. We can consequently state that the suspensory ligament is represented by three blades: two superficial blades sharing the role of the fundiform ligament and a deep blade on the midline, which is continued by the deep fascia of the penis, thus keeping the penile body on the median line [4, 5, 15, 20, 21].

**Fig. 11:**
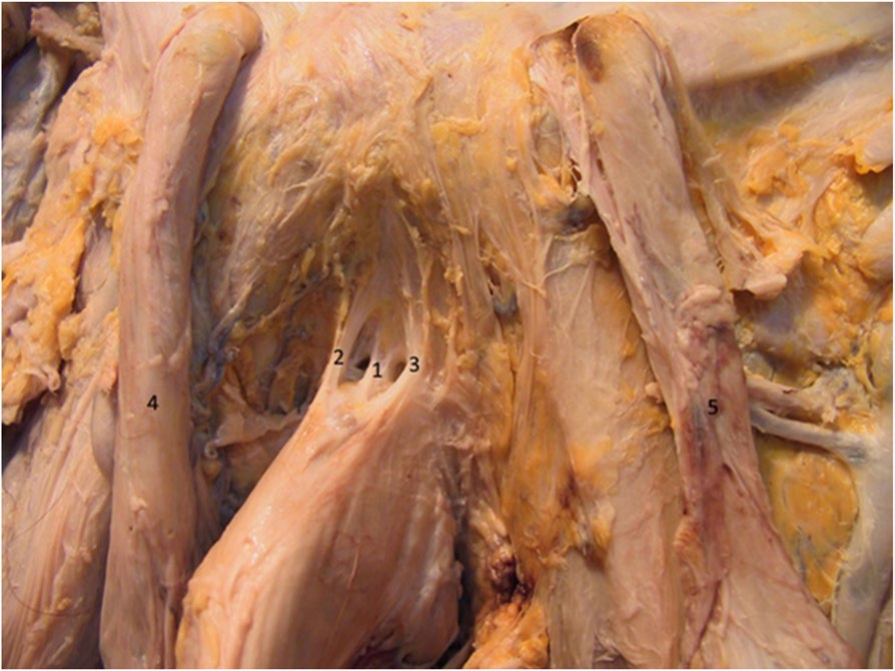
Median blade of the suspensory ligament – detail. 1. Median bundle of suspensory ligament. 2,3. Lateral bundles of the suspensory ligament. 4. Right spermatic cord. 5. Left spermatic cord

Figure 11 confirms the presence of the median bundle of the suspensory ligament. We can observe that the two lateral blades of the suspensory ligament fuse anteriorly on the median line and inferiorly continue their pathway around the penile body. The space found between them (the cavum) contains the median bundle of the suspensory ligament and the dorsal neurovascular bundle of the penis [6, 9, 17, 21].

**Fig. 12:**
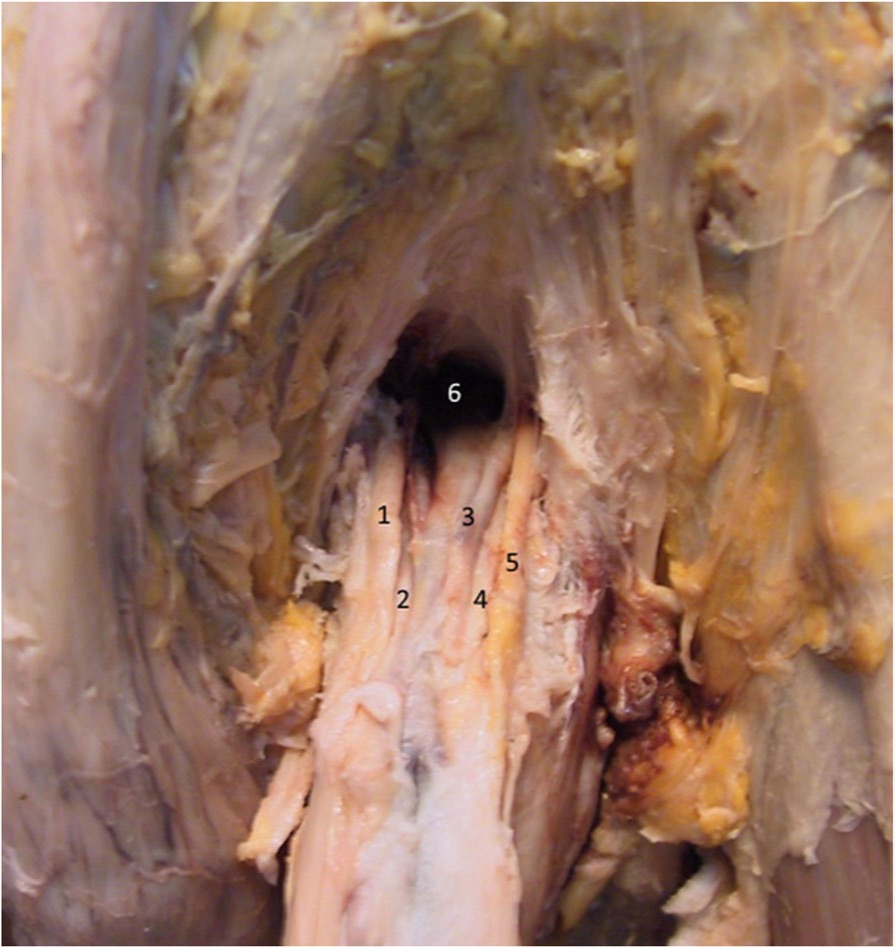
Dorsal neurovascular bundle of the penis and the space between the symphysis and the penis. 1. Right dorsal nerve. 2. Right dorsal artery. 3. Deep dorsal vein. 4. Left dorsal artery. 5. Left dorsal nerve. 6. Subpubic space

After the complete removal of the two ligaments and of the deep fascia of the penis, the dorsal neurovascular bundle becomes apparent in Fig. 12 [15, 16], where the space between the penis and the symphyseal arch is highlighted. This space may represent a communication between the prepubic and the intrapelvic regions [3]. The Luschka ligament’s fibers were resected [18]. In practice, the riskiest time in the augmentation technique is represented by the resection of the median blade of the suspensory ligament, its inferior attachment being in direct relation with the penile dorsal vascular-nervous bundle [5, 9, 12, 16, 21].

The issue of maintaining the penile body in position whilst also keeping it on the midline during penile aesthetic surgeries is solved in a complex way. Both the fundiform and the suspensory ligaments connect the penis to the pelvic wall. The terminal fibers of these ligaments surround the penile body in a hammock-like manner and create the presymphyseal curvature of the penis. However, the median bundle fibers of the suspensory ligament that originate on the inferior aspect of the symphyseal arch end directly on the deep penile fascia and keep the penile body on the median line. The possibility of damaging either the external pudendal vessels (located at the base of the fundiform ligament) or the dorsal neurovascular bundle of the penis (located at the base of the suspensory ligament) represents a real surgical risk in ligamentolysis.

The space between the penis and the symphyseal arch is closed from superficial to deep by the divergent bundles of the fundiform ligament, the lateral blades of the suspensory ligament and the median bundle of the suspensory ligament. This space is also limited by the connective fibers system that early anatomists called the Luschka ligament [22].

## Conclusions

It is obvious that if the suspensory apparatus is sectioned, this morphofunctional unit located between the penis and the pelvic wall loses its function and the movements of the penile body no longer faithfully follow the movements of the pelvis. Furthermore, this can also result in changes in the anterior curvature of the penis.

Regarding penile augmentation following the sectioning of the suspensory apparatus, we state that the relative penile elongation is the result of the decrease of the penile presymphyseal curvature. The possible aesthetic benefit is drastically impaired by the dynamic functional disadvantage, as mentioned above. The main surgical risk is the injury of the external pudendal vessels or of the dorsal vascular bundle of the penis, in which case, a local hematoma may form and subsequently migrate inside the pelvis, through the space between the dorsal aspect of the penis and the symphyseal arch.

## Data Availability

The cadavers used in this study were provided by the Anatomy Discipline of the Morphology Department of the University of Medicine and Pharmacy Bucharest. The ethics committee of the “Victor Babes” Diagnosis and Treatment Center approved the utilization of pelvic MRI examination In this study.
